# ApoM is an important potential protective factor in the pathogenesis of primary liver cancer

**DOI:** 10.7150/jca.53115

**Published:** 2021-06-04

**Authors:** Yaping Bai, Wenjun Pei, Xiao Zhang, Huihao Zheng, Changchun Hua, Jiao Min, Lisheng Hu, Shuangqiu Du, Zuyue Gong, Jialin Gao, Yao Zhang

**Affiliations:** 1 Anhui Province Key Laboratory of Biological Macro-molecules Research, Wannan Medical College, Wuhu 241002, China.; 2 Department of Biochemistry and Molecular Biology, Wannan Medical Collage, Wuhu 241002, China.; 3 Department of Pediatric Surgery, The First Affiliated Hospital Of USTC-Division of Life sciences and medicine, University of China, Hefei 230001, China.; 4 Department of endocrine, The First Affiliated Hospital of Wannan Medical College, Wuhu 241002, China.

**Keywords:** apolipoprotein M, protective factor, primary liver cancer

## Abstract

In recent years, abnormal liver lipid metabolism has emerged as one of the important pathogenesis pathways of primary liver cancer. It is highly important to identify the mechanisms to explore potential prevention and treatment targets. Apolipoprotein M is specifically expressed in the liver and participates in liver lipid metabolism, but the evidence that ApoM affects primary liver cancer is insufficient. The Cancer Genome Atlas (TCGA) database and clinical case analysis, as well as animal level and cell level analysis suggest that the expression level of *ApoM* gene in cancer tissues is lower than that in paracarcinoma tissues. Further experimental research found that the deletion of ApoM significantly increased the proliferation of mouse liver cancer cells (Hepa1-6) and inhibited the level of apoptosis induced by cisplatin. In addition, mouse liver cancer cells lacking ApoM showed stronger migration and invasion capabilities in transwell experiments. In contrast, overexpression of ApoM in Hepa1-6 cells and Huh-7 cells showed an inhibition of proliferation, up-regulation apoptosis and reduced migration and invasion. *In vivo*, the deletion of the *ApoM* accelerated tumorigenesis in nude mice and allowed the mice to develop liver tumor mutations more quickly under the induction of N-nitrosodiethylamine and the survival time of mice was shorter than that control. Therefore, ApoM may be a potential protective factor to inhibit the occurrence and development of primary liver cancer.

## Introduction

Primary liver cancer is one of the most common malignant tumors, with a high mortality rate worldwide, and its incidence is increasing year by year 1. In view of its high degree of malignancy and often undetected development, most patients have entered the middle and advanced stages when diagnosed. Therefore, the study of the pathogenesis of primary liver cancer is a major scientific issue that needs to be resolved urgently. Previous studies have shown that the alcoholism, smoking, chronic viral hepatitis, liver cirrhosis, and aflatoxin are risk factors for liver cancer 2. However, not all patients who present with primary liver cancer have these risk factors, and so it is possible that some other important factors have been ignored. Metabolic disorders, especially lipid metabolism disorders, are an important microenvironment for tumor pathogenesis. At present, the “metabolic disease” theory is being applied in relation to the onset of primary liver cancer 3, 4. Changes in liver lipids metabolism are closely related to the occurrence of liver cancer, and non-alcoholic fatty liver disease may be identified as one of the main causes of primary liver cancer in the future 5.

As a member of the apolipoprotein family, Apolipoprotein M (ApoM) has been shown to be involved in related processes of lipid metabolism. Research on ApoM has mostly focused on atherosclerosis. For example, the HDL-ApoM-S1P complex can protect the vascular endothelium and has a certain anti-atherosclerotic activity 6, 7. In addition, our previous studies have shown that autophagy dysfunction caused by ApoM deficiency is an important factor in liver steatosis 8. Is it now appropriate to think about the relationship between ApoM and liver cancer? Other studies have shown that miRNA-573, which directly targets the negative regulation of *ApoM*, can promote tumor cell proliferation by inhibiting the apoptosis of liver cancer cells *in vitro* 9. However, it is worth noting that the development of liver cancer in the body is a long and complicated process, during which changes in the environment in the body cannot be ignored.

There are many factors that affect the formation and development of tumors, mainly changes in the levels of tumor cell proliferation, apoptosis, migration, and invasion caused by changes in the physical and chemical environment of the body 10, 11. Tumor cells have unlimited ability to proliferate and divide. This abnormal proliferation may be closely related to the disorder of the tumor cell cycle, which is a typical biological feature of cancer 12, 13. Apoptosis is a method of programmed cell death regulated by genes, and it is an essential function the body uses to maintain normal physiological homeostasis 14. In malignant tumor cells, the apoptosis process is blocked and the cell cycle regulation mechanism is destroyed, so that the rapid and massive proliferation of tumor cells is characteristic of cancer. To date, many postoperative chemotherapy drugs for cancer have been developed based on the apoptosis mechanism 15, 16. The migration and invasion capabilities of tumor cells also determine the outcome of the disease, and which induced metastasis really threatens patients and troubles doctors 17, 18.

Considering all of these factors, it is reasonable to suspect a connection between ApoM and primary liver cancer. Therefore, it is important to analyze whether the* ApoM* gene is involved in the occurrence and development of liver cancer. This study systematically explored the role of ApoM in the growth of primary liver cancer cells including the aspects of proliferation, apoptosis, migration, and invasion. In addition, the influences of the *ApoM* gene on the initiation of liver cancer were aslo examined by observing the rate of tumor formation *in vivo* model induced by N-nitrosodiethylamine. This work will add a new basis to the theory of the correlation between apolipoprotein and liver tumors, and further enrich functional research on apolipoprotein.

## Materials and methods

### TCGA database screening

Software: R (version 3.6.3) (statistical analysis and visualization); R package: mainly ggplot2 (for visualization); Statistical method: Use paired sample T test; Molecule: APOM [ENSG00000204444]; Data: TCGA (https://portal.gdc.cancer.gov/) LIHC (hepatocellular carcinoma) project RNAseq data in level 3 HTSeq-FPKM format; Data filtering: keep paired samples; Data conversion: RNAseq data in FPKM (Fregments Per Kilobase per Million) format is converted to log2 and then compared between samples; Significance mark: ns, *p*≥0.05; *p<0.05; **p<0.01; ***p<0.001.

### Clinical pathology sample

The clinical cases of liver cancer collected in the study were all from the Pathology Bank of the First Affiliated Hospital of Wannan Medical College, and all pathological samples were subject to the informed consent of the patients.

### Cell culture

Primary liver cancer cells Hepa1-6 and Huh-7 (Pronox, Wuhan) are in high-glycemic DMEM (GIBCO, USA) containing 10% fetal bovine serum (ExCell, AUS) and 1% P/S (GIBCO, USA). Mouse normal hepatocyte AML12 (ATCC, United States) cells are contained in 10% fetal bovine serum (ExCell, AUS), 1% Insulin-transferrin-sodium (Sigma, USA) and 0.02% dexamethasone (Sigma, USA) RPMI-1640 culture solution (GIBCO, USA), and incubate at 37 °C in an environment with 5% CO_2_.

### Immunohistochemistry (IHC) staining

Twenty-three clinical liver cancer samples were collected from the pathology bank of Yijishan Hospital, Wannan Medical College, for paraffin sectioning. After antigen recovery and staining, ImageJ was used to count the average density (AOD) of ApoM in cancerous tissues and adjacent tissues, and pathological analysis of 23 clinical specimens was performed to perform statistics on gender, age, presence or absence of masses, liver cirrhosis, and necrosis.

### Crispr/Cas9 vector construction and infection

The *ApoM* gene deletion model of Hepa1-6 and Huh-7 cells was obtained by designing sgRNA sequence, packaging and infecting lentivirus. This sgRNA sequence: Hepa1-6-ApoM^-/-^F: CACCGAAAAGTTGCCAACTCTTCCG, R: AAACCGGAAGAGTTGGCAACTTTTC; Huh-7-ApoM^-/-^F: CACCGTCAGGTGGTAGATCCATTTC, R: AAACGAAATGGATCTACCACCAGGC were designed and obtained through the online website ccTop.com and connected to the Lenticrispr-v2, Lenticrispr-dual-puro vector. The CDS region of ApoM was amplified and ligated into the PECMV-3xflag-c vector to package the lentivirus to obtain an overexpression model of the Hepa1-6-*ApoM* gene. The primers were: Hepa1-6-ApoM-OP-F: CGGGATCCCCACCATGTTCCACCAAGTTTGGGCAG, R: CCCTCGAGCTTGCTGGACAGCGGGCAGGCCT.

### Edu experiment

Edu kit was purchased from Guangzhou Ruibo (C10310-1). Each group of logarithmic growth phase cells was taken, reagent A was added, incubated for 2 hours, fixed with 4% paraformaldehyde, incubated with 2 mg/mL glycine for 5 minutes, washed with PBS, added 0.5% TritonX-100 PBS penetrant, incubated for 10 minutes, and washed with PBS once. Reagent B was added and incubated for 30 minutes; washed with penetrant twice, incubated with 1X Hoechst 33342 reaction solution for 0.5 h, washed with PBS, and observed and analyzed with fluorescence inverted microscope (Olympus, Japan) 10×10.

### Cell cycle detection

Cells were collected according to the instructions of the cell cycle detection kit (China KGI, Cat: KGA105-KGA108), washed with PBS (2000 rpm, 5 min), prepared in a single cell suspension, centrifuged, and removed from the supernatant; 70% ice ethanol was added to the cells and fixed overnight at 4 °C. The supernatant was discarded after centrifugation, 1:9Rnase A:PI staining working solution was added and incubated for 30 min at room temperature in the dark. Flow cytometer (BD, USA) was used to detect.

### Flow cytometry

Cells were collected according to the instructions of the Apoptosis Detection Kit (BD, USA, Cat: 556547), then resuspended in pre-cooled 1×PBS and washed twice. 300 μL of 1×Binding buffer was added to resuspended cells, and then 5 μL of AnnexinV-FITC to mix well (BD, USA) was added and incubated at room temperature in the dark for 15 minutes. PI label was added for 5 minutes before using the machine, with 200 μL of 1×Binding buffer.

### Western blotting

The cells were dissolved in RIPA lysis buffer and PMSF, and the protein concentration was measured with an ND2000 micro-spectrophotometer. Proteins were separated by SDS-PAGE (8% or 15% gels), transferred to PVDF membranes, and incubated with the primary antibody overnight. The next day, the membranes were incubated with the corresponding secondary antibody at room temperature for 2 hours. ECL chemiluminescent fluid and imaging system were used for exposure and data analysis. Primary antibody used: ApoM(China, ABclonal/A5336 1:1000), Caspase 3(UK, abcam/ab13847 1:1000), Caspase 9(UK, abcam/ab69514 1:1000), Bax(US, CST/2772S 1:1000), Bcl 2(US, CST/3498S 1:1000), MMP-2 (China, BTL/BTL2798T 1:1000), β-actin(US, Sigma/A1978 1:5000) related protein expression levels.

### Transwell migration and invasion experiments

Cells of each group were cultured to the logarithmic growth phase. After digestion and centrifugation, the cells were resuspend them in serum-free medium. We took 200 μL of serum-free cell suspension (1×10^5^ cells) of each group into the upper chamber of Transwell chamber, added 600 μL of DMEM medium containing 20% FBS to the lower chamber, and incubated it in a 5%CO_2_ cell incubator. After incubation at 37 °C for 24 h, the culture plate was taken out, washed with PBS once, fixed with 4% paraformaldehyde for 30 minutes, then discarded the solution, washed again. The migrated cells were stained with 1% crystal violet solution for 30 minutes. Under a fluorescent inverted microscope, images were captured in 5 fields randomly to count the number of cells penetrating the membrane randomly. The Matrigel glue needed to be taken out from -20 °C in a refrigerator and put at 4 °C overnight, The next day, we diluted the Matrigel glue with serum-free cell culture medium to 200 μL/ml at 4 °C, added 50 μL Matrigel on the Transwell polycarbonate membrane, and place the culture plate in the 37 °C 5% CO_2_ cell incubator. After 2 hours, a basement membrane structure was formed. Then we added 600 μL of 20% FBS DMEM culture medium to the basolateral side and 200 μL of serum-free culture medium to the inside. The remaining steps were the same as the migration experiment.

### Tumor formation experiment in nude mice

The mouse liver cancer Hepa1-6-control and Hepa1-6-ApoM^-/-^ cells cultured to the logarithmic growth phase were adjusted to 1×10^7^/ml. The cells were injected into the left armpit of a 5-week-old BALB/c nude mouse (Nanjing Jicui Yaokang Biotechnology Co). The tumor diameter of the nude mouse is measured every 4 days. After 20 days, we sacrificed the mouse by cervical dislocation and taken the tumor in -80 °C for follow-up tests.

### Induction of liver cancer experiment in mice

We weekly used N-nitrosodiethylamine solution (Shanghai Macleans Biochemical 35 mg/kg) intraperitoneal injection of randomly selected 8-week-old healthy 30 C57BL/6J male WT and 30 *ApoM^-/-^* mice (Shanghai Southern Model Biology Research Center) and after injection At 0, 4, 8, 12, 16 and 20 weeks, 3 mice in each group were randomly selected to be anesthetized with 10% urethane. After weighing, blood was taken from the inner canthus for liver function test, the abdominal cavity was opened, and the whole liver was taken out. After recording, we stored it at -80 °C for subsequent testing.

### Statistical analysis

GraphPad Prism 8.0 statistical software was used for data analysis. The experiments were repeated three times. The experimental data were expressed as mean ± standard deviation (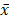 ± s). The t-test was used to compare the data between the two groups. *P*<0.05 was considered statistically significant.

## Results

### The expression level of ApoM in cancer tissues is lower than that in adjacent tissues

**Figure 1:**
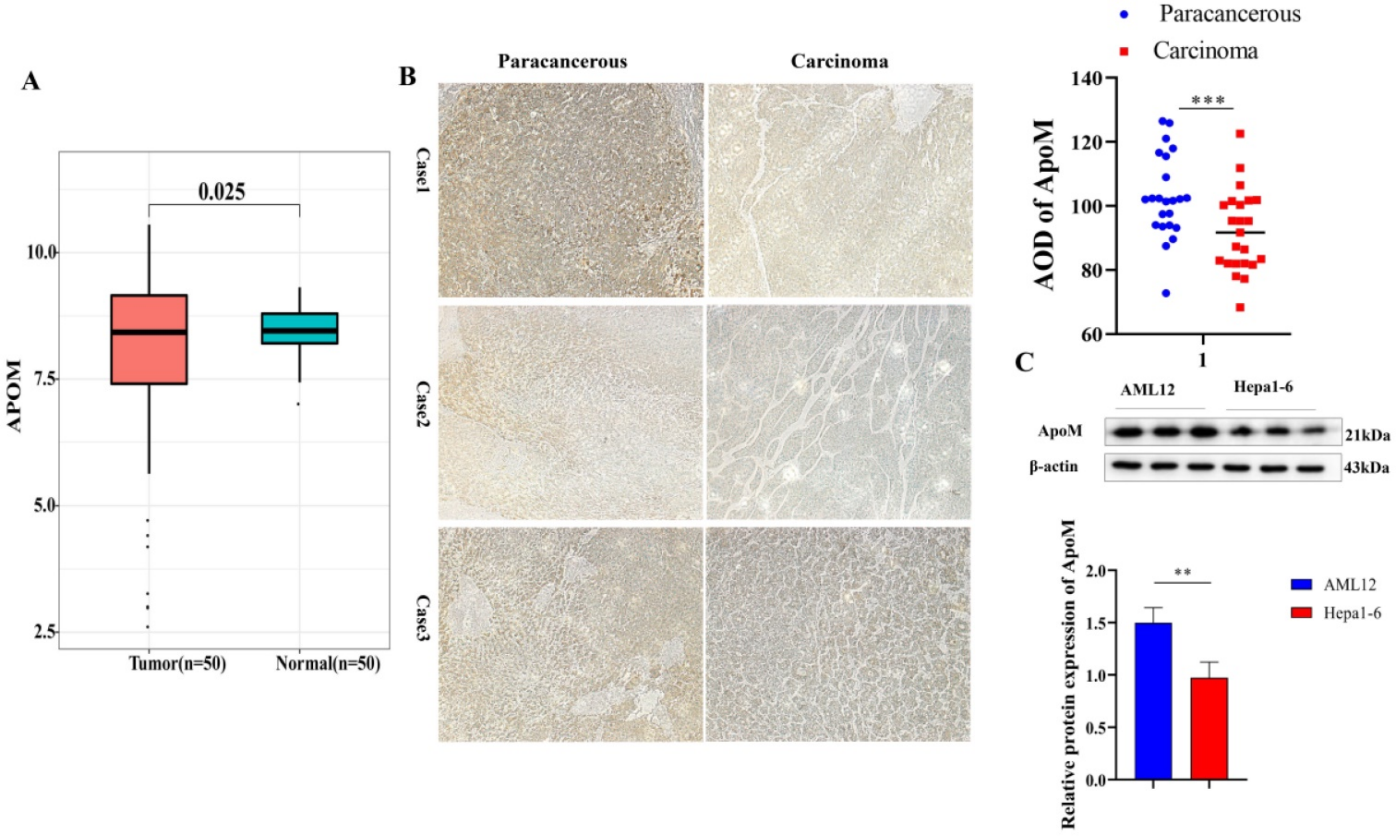
** The expression level of ApoM in cancer tissues is lower than that in adjacent tissues. (A)** 50 matched samples of primary liver cancer were selected from the TCGA database. The analysis showed that the expression level of *ApoM* gene in cancer tissues was lower than that in paracarcinoma tissues, and the difference was statistically significant (*P*=0.025). **(B)** Using immunohistochemical method to detect the expression level of ApoM in cancer tissues and adjacent tissues of liver cancer patients, the expression level of ApoM in cancer tissues decreased significantly (*P*=0.0063). **(C)** The expression level of ApoM in the normal mouse liver cell line AML12 was higher than that in the mouse liver cancer cell line Hepa1-6 (*P*=0.012) *p<0.05, **p<0.03, ***p<0.01.

To initially verify whether there is a connection between ApoM and primary liver cancer, 50 matched samples of primary liver cancer tissues were selected from the TCGA database and analyzed. The expression level of the *ApoM* gene in the cancer tissues was lower than that in paracarcinoma tissues (Fig. 1A). another 23 clinical specimens of liver cancer were collected for verification, which was consistent with the results of bioinformatics analysis (Fig. 1B); at the same time, Compared with the normal mouse liver cell line AML12, Hepa1-6 showed a lower ApoM expression level (Fig. 1C).

### Expression level of the* ApoM* gene affects the proliferation of liver cancer cells

**Figure 2:**
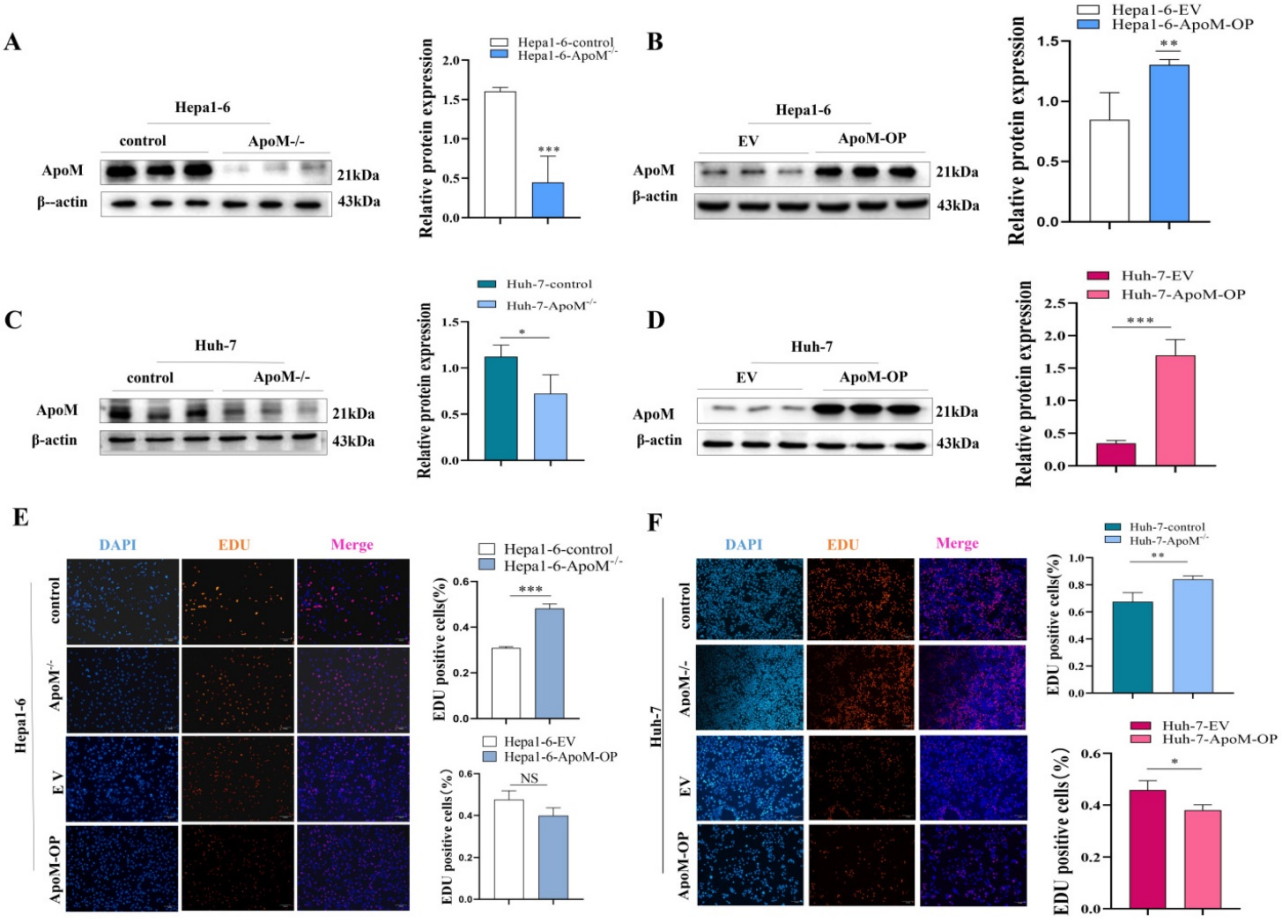
** Expression level of the *ApoM* gene affects the proliferation of liver cancer cells. (A,B)** Western blot method verified the knockout of the *ApoM* gene in the Hepa1-6 cell line (*P*=0.00049) and the overexpression model (*P*=0.015) were successfully constructed. **(C,D)** Western blot method verified the knockout of the *ApoM* gene in the Huh-7 cell line (*P*=0.044) and the overexpression model (*P*=0.0000070) were successfully constructed. **(E)** Edu proliferation experiment showed that the cell proliferation activity of *ApoM* gene deletion group increased (*P*=0.00050), and *ApoM* gene was overexpressed the cell proliferation activity had a downward trend (*P*=0.070). **(F)** The Edu proliferation experiment showed that the cell proliferation activity of the *ApoM* gene deletion group increased (*P*=0.017), while the cell proliferation activity of the* ApoM* gene overexpression showed a downward trend in Huh-7 cells (*P*=0.032). 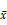
± s, n≥3, *p<0.05, **p<0.03, ***p<0.01.

The level of proliferation activity is one of the evaluation indicators of cell growth status. We constructed a mouse liver cancer cell model with *ApoM* gene deletion and overexpression and verified it (Fig. 2A, 2B). Edu experiments were used to determine whether the expression level of the* ApoM* gene affects the proliferation of liver cancer cells. The results showed that, compared with the control, the deletion of the *ApoM* caused an increase in cell proliferation rate, while it was decreased in overexpression of ApoM (Fig. 2E). In addition, consistent results were obtained in the human liver cancer cell line Huh-7. The deletion of* ApoM* gene promotes its proliferation, while the overexpression of *ApoM* gene inhibits proliferation (Fig. 2C, 2D, 2F).

### *ApoM* gene inhibits tumor formation and the liver cancer cell cycle

**Figure 3:**
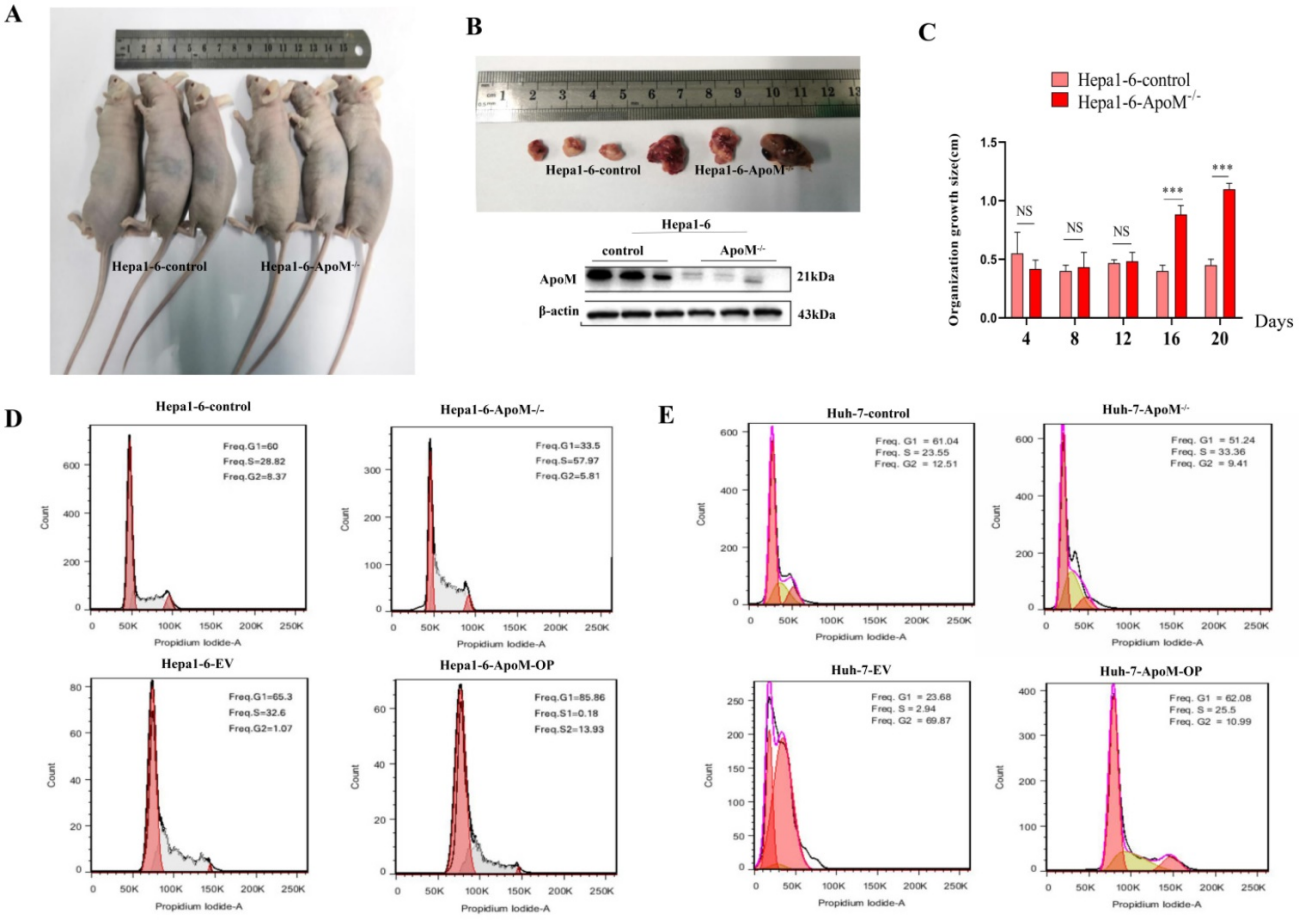
***ApoM* gene inhibits tumor formation rate and liver cancer cell cycle in nude mice. (A)**The tumor formation experiment in nude mice showed that the same number of cells were injected subcutaneously in the two groups. Compared with the tumors in the Hepa1-6-control group, the tumors grew faster in Hepa1-6-*ApoM^-/-^*. **(B)**The *ApoM^-/-^* model in nude mouse tumors was successfully constructed by Western blot analysis. **(C)**The tumor growth size of nude mice showed that in the sixteenth day, the tumor in the Hepa1-6-*ApoM^-/-^* group was larger than that in the Hepa1-6-control group (*P*=0.00078). **(D)** Using cell cycle experiments to analyze the *ApoM* gene deletion group, the sum of G0/G2 and S phase is larger, and the proliferation rate is faster; the* ApoM* gene overexpression group, the G0/G1 phase is blocked and the proliferation rate is slower. **(E)** Similarly, the cell cycle test of Huh-7-*ApoM^-/-^* model and Huh-7-*ApoM*-OP model is consistent with the above conclusions.

In order to verify the *in vitro* experiment, we carried out *in vivo* experiments investigating tumor formation in BALB/c nude mice. Significantly different from the control group, the *ApoM* gene deletion group had a faster tumor growth rate (Fig. 3A), and we found that the difference between the two appeared on the 16th day after subcutaneous injection (Fig. 3C). The tumor tissue was removed to verify the expression level of ApoM protein (Fig. 3B), and we thus determined that the expression level of ApoM affected the proliferation of liver cancer cells. The results of cell cycle experiments suggested that, in liver cancer cells with *ApoM* gene deletion, the number of cells in the G0/G1 phase was reduced, and the proportion of cells in the S+G2/M phase was higher; in contrast, upon overexpression of *ApoM*, the G0/G1 phase was prolonged. Hence the decrease in the proliferation activity of the cells in the overexpression group was probably caused by blockage of the G0/G1 phase (Fig. 3D). Similarly, the cell cycle test of Huh-7-*ApoM^-/-^* model and Huh-7-*ApoM*-OP model is consistent with the above conclusions (Fig. 3E).

### *ApoM* gene promotes the apoptosis of liver cancer cells

**Figure 4:**
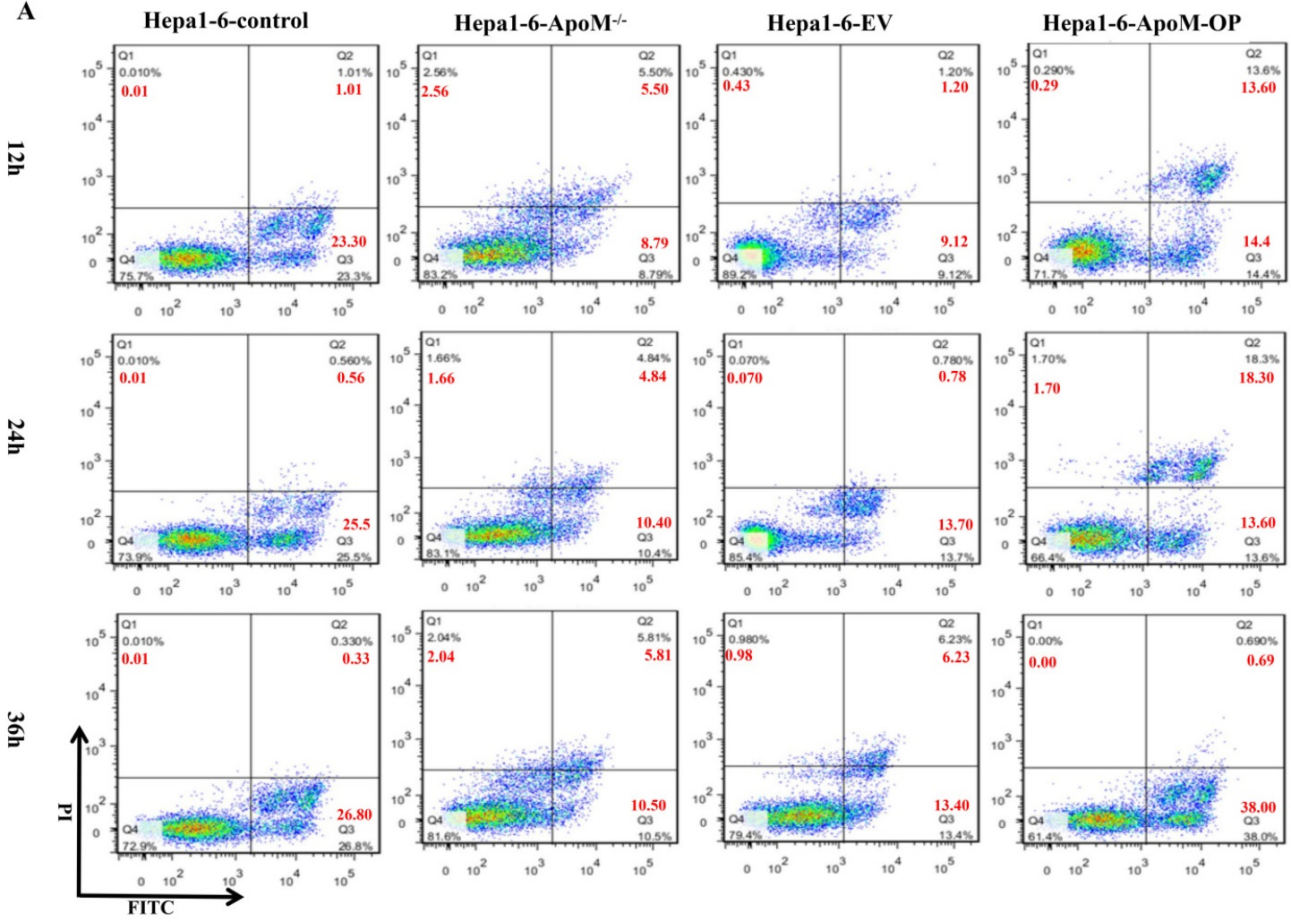
***ApoM* gene promotes apoptosis of liver cancer cells. (A)** Flow cytometry analysis showed that the apoptotic level of cells transfected with *ApoM^-/-^* lentivirus changed after 12h, 24h and 36h. In Hepa1-6 cells, the apoptosis rate of the *ApoM* gene deletion group was lower than that of the control group; while the cells transfected with *ApoM* overexpressing lentivirus were 12 hours, 24 hours and 36 hours later, the cells of the Hepa1-6-*ApoM*-OP group The apoptosis rate was higher than that of the EV group.

Flow cytometry analysis showed that, in the Hepa1-6-control group, the proportions of early apoptotic cells and late apoptotic cells were 23.3% and 1.01%, respectively, at 12 h after transfection, 25.50% and 0.56%, respectively, at 24 h after transfection, and 26.80% and 0.33%, respectively, at 36 h after transfection. In the *ApoM* gene deletion group, the proportions of early apoptotic cells and late apoptotic cells were 8.79% and 5.50%, respectively, 12 h after transfection, 10.40% and 4.84%, respectively, 24 h after transfection, and 10.5% and 5.81%, respectively, 36 h after transfection. Therefore, the *ApoM* gene deletion group had a lower level of apoptosis than the control group (Fig. 4A). In contrast, in the Hepa1-6-EV group, the proportions of early apoptotic cells and late apoptotic cells were 9.12% and 1.20%, respectively, at 12 h after transfection, 13.70% and 0.78%, respectively, at 24 h after transfection, and 13.40% and 6.23%, respectively, at 36 h after transfection. In the Hepa1-6-*ApoM*-OP group, the proportions of early apoptotic cells and late apoptotic cells were 14.40% and 13.60%, respectively, at 12 h after transfection, 13.60% and 18.30%, respectively, at 24 h after transfection, and 38.00% and 0.69%, respectively, at 36 h after transfection. These results indicated that the level of apoptosis increased after overexpression of *ApoM* (Fig. 4A).

### *ApoM* gene promotes the expression of key apoptotic proteins

**Figure 5:**
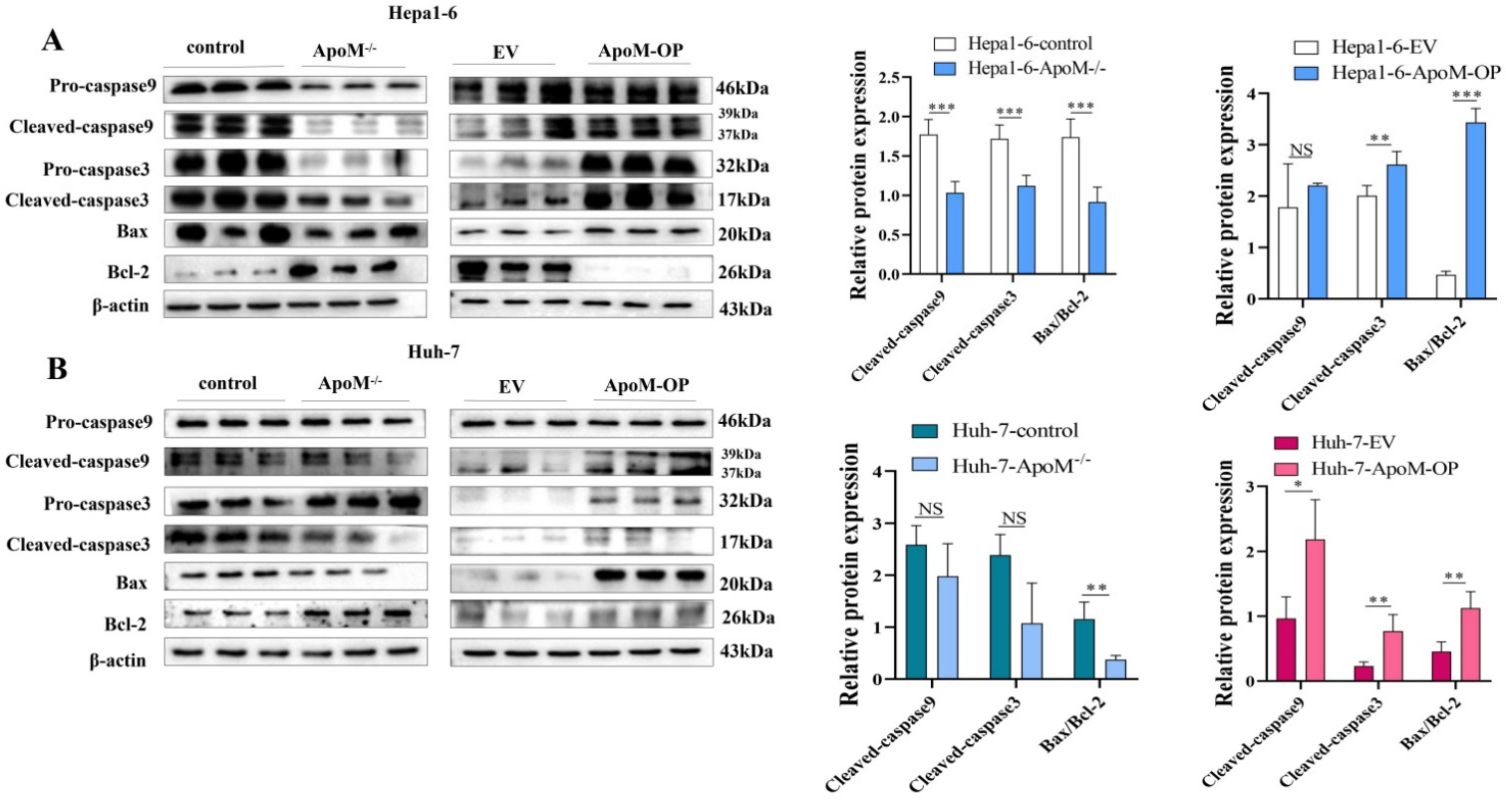
***ApoM* gene promotes the expression of key apoptotic proteins. (A, B)** Western blot analysis of the expression levels of key apoptotic proteins, the results showed that the deletion of *ApoM* gene reduced the expression levels of Cleaved-caspase-3, Cleaved-caspase-9 and Bax/Bcl-2 cell apoptosis-related proteins. The overexpression of *ApoM* gene increased the expression of Cleaved-caspase-3, Cleaved-caspase-9 and Bax/Bcl-2 apoptosis-related proteins, and statistical analysis was performed. *p<0.05, **p<0.03, ***p<0.01.

According to the flow cytometry results, we investigated further down to the molecular level. In the *ApoM* gene deletion group, the expression levels of Cleaved-caspase-3, Cleaved-caspase-9 and Bax/Bcl-2 apoptosis-related proteins decreased. In the *ApoM* overexpression group, the expression of Cleaved-caspase-3, Cleaved-caspase-9 and Bax/Bcl-2 apoptosis-related proteins increased (Fig. 5A, 5B).

### The deletion of *ApoM* gene aggravate the migration and invasion in liver cancer cells

**Figure 6:**
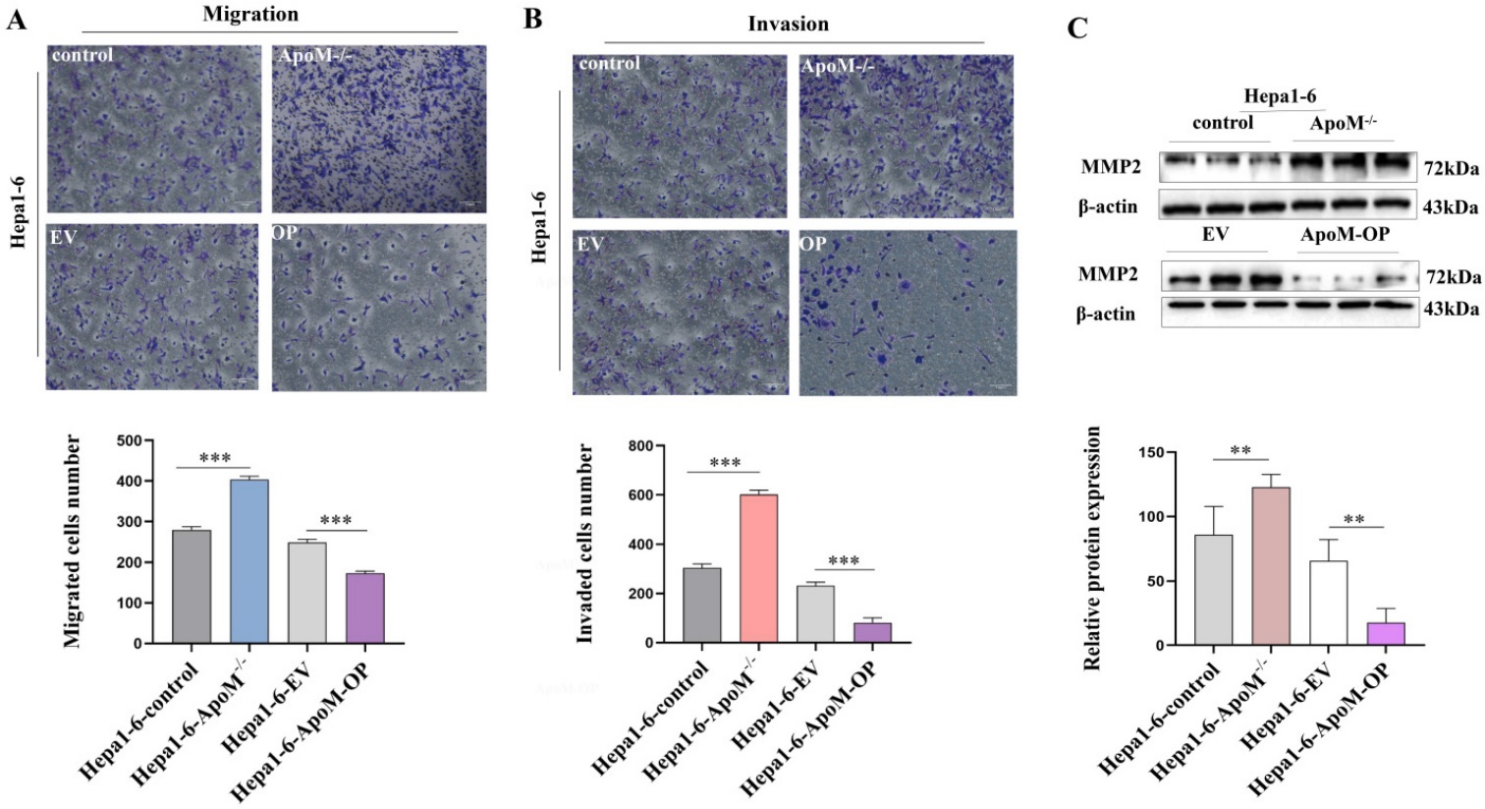
** The deletion of *ApoM* gene aggravate the migration and invasion of Hepa1-6 liver cancer cells. (A)** The Transwell migration experiment indicated that the *ApoM* gene deletion group had stronger migration ability than the control group (*P*=0.000044), while the overexpression group decreased (*P*=0.00018). **(B)** The Transwell invasion experiment indicated that the *ApoM* gene deletion group had stronger invasion ability (*P*=0.000025), while the overexpression group decreased (*P*=0.00042). **(C)** Western blot analysis was used to analyze that the *ApoM* gene deletion group had a higher MMP-2 protein expression level (*P*=0.027), and the overexpression group was the opposite (*P*=0.027). All data were 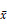
± s of 3 independent measurements, n≥3, *p<0.05, **p<0.03, ***p<0.01.

Studies have found that many patients with primary liver cancer die of liver cell metastasis. Transwell experiments were used to detect the migration and invasion abilities of cells in each group. The results suggested that the *ApoM* gene deletion group had stronger migration and invasion capabilities than the control group, while the overexpression group had decreased migration and invasion capabilities (Fig. 6A, 6B). At the same time, Western blot results showed that, compared to the control group, the *ApoM* gene deletion group had a higher level of MMP-2 protein expression (Fig. 6C) whereas the overexpression group had a lower level (Fig. 6C), suggesting that the *ApoM* gene inhibited the migration and invasion of liver cancer cells.

### Deletion of the* ApoM* gene accelerates the rate of liver cancer formation in mice treated of N-nitrosodiethylamine induction

**Figure 7:**
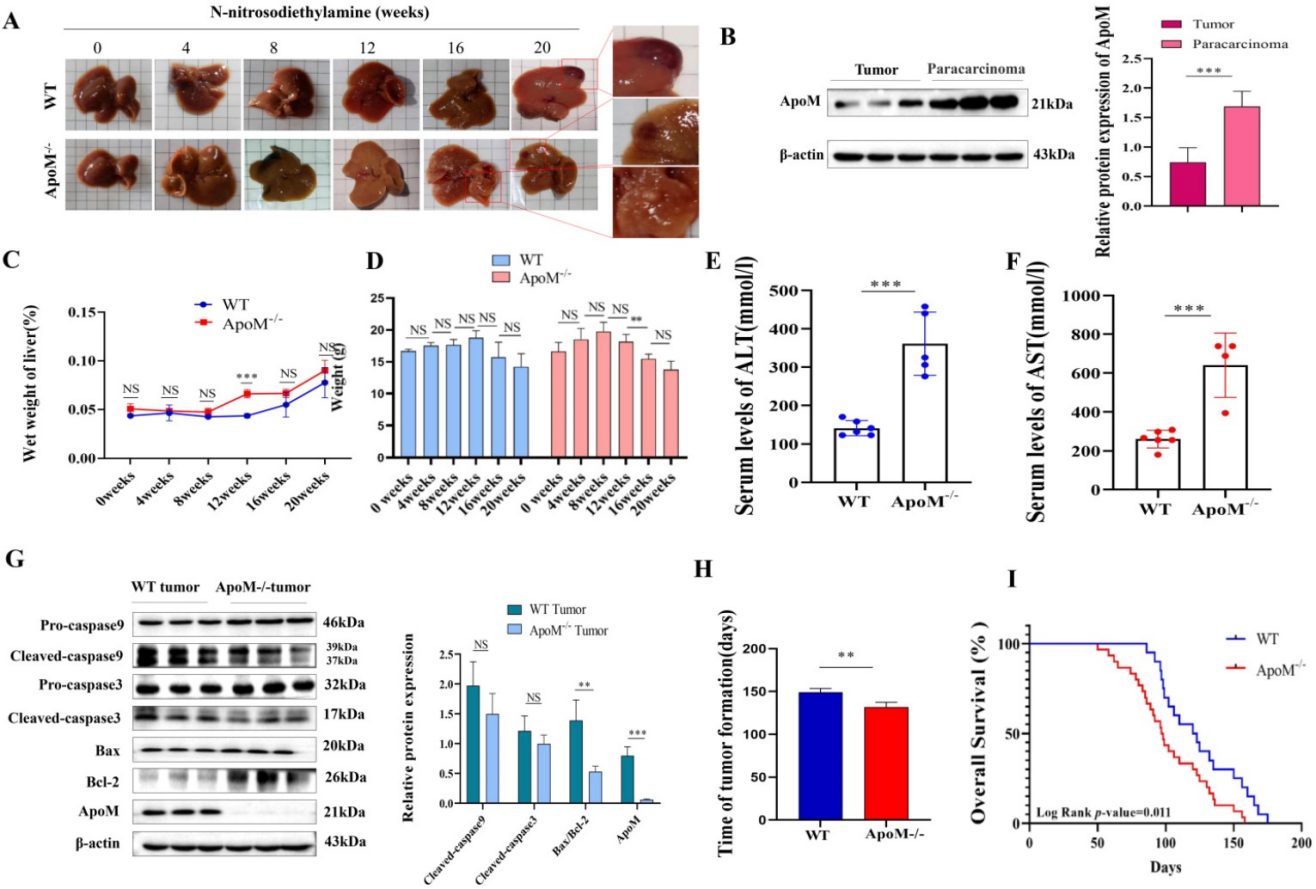
** The deletion of *ApoM* gene accelerates the tumorigenesis rate of C57BL/6J mouse liver cancer induced by N-nitrosodiethylamine. (A)** Livers were taken at 0, 4, 8, 12, 16 and 20 weeks. Compared with WT mice, *ApoM* gene-deficient mice developed tumors earlier under N-nitrosodiethylamine induction. **(B)** Western blot assay showed that the ApoM level in the cancer tissue of WT mice in the liver cancer induction model group was lower than that in the paracarcinoma tissues (*P*=0.010). **(C)** Compared with the WT group, the* ApoM* gene deletion group had higher wet liver weight per month. At 12 weeks, the wet liver weight of the *ApoM* gene deletion group was higher than that of the WT group and was statistically significant (*P*=0.0048). **(D)** The body weight of mice in the WT group increased from 0 weeks to 12 weeks, and their body weight decreased after 12 weeks. The body weight of mice in the *ApoM* gene deletion group increased from 0 to 8 weeks, and their body weight decreased after 8 weeks. **(E, F)** Liver function ALT (*P*=0.00013) and AST (*P*=0.00061) levels of liver cancer induction model *ApoM^-/-^* group are higher than those of WT group, and have statistical significance. **(G)** Western blot analysis of liver cancer induction model, the key apoptosis proteins Cleaved-caspase9 (*P*=0.20), Cleaved-caspase3 (*P*=0.26), Bax/Bcl-2 (*P*=0.014) in wild mice were higher than *ApoM* gene deletion group. (*P*=0.0011). **(H)** Compared with the WT group, the *ApoM* gene deletion group has a faster time for liver tumors, which was statistically significant (*P*=0.028). **(I)** The survival of *ApoM^-/-^* mice was shorter than that of WT mice (*P*=0.011).

WT and *ApoM* gene-deficient mice were intraperitoneally injected with 35 mg/kg N-nitrosodiethylamine solution to induce liver cancer models. At the end of 0, 4, 8, 12, 16, and 20 weeks, the mice were killed and the livers were taken to determine the *ApoM* gene pair and the effect of tumor formation rate in C57BL/6J mice. Compared with WT mice, *ApoM* gene-deficient mice developed liver tumors earlier (Fig. 7A, 7H). Throughout the induction process, At 12 weeks, the liver wet weight of the *ApoM* gene-deficient group was higher than that of the WT group and was statistically significant (Fig. 7C); from the 12^th^ week to the 16^th^ week, the weight of the *ApoM* gene-deficient mice decreased significantly (Fig. 7D). The expression level of ApoM in cancer tissues of WT mice was significantly lower than that of paracarcinoma tissues (Fig. 7B). Liver function ALT and AST levels of liver cancer induction model *ApoM^-/-^* group were higher than those of WT group (Fig. 7E, 7F). In addition, the level of apoptosis in cancer tissues of WT mice was higher than that of *ApoM^-/-^* mouse cancer tissues (Fig. 7G). Finally, the results of the survival curve indicated that the survival time of *ApoM^-/-^* mice was shorter than that of WT mice (Fig. 7I).

## Discussion

To date, many apolipoproteins have been listed as tumor detection indicators. For example, some previously discovered apolipoproteins such as ApoB, ApoA1, and ApoE have been included in the biochemical tests of liver function and blood lipids^19^, ^20^. Compared with these apolipoproteins, ApoM was discovered late. Considering its distribution across various tissues in mice and humans, the expression level of the ApoM in the liver is by far the highest. As one of the pivotal organs of metabolism, the liver is vital in maintaining the steady state of glucose and lipid metabolism. As mentioned above, the occurrence and development of liver cancer are inseparable from abnormal lipid metabolism 21, 22. In the future, non-alcoholic fatty liver disease will even become the main cause of liver cancer 23, 24. And our previous studies have shown that the decline of *ApoM* gene expression level can promote the accumulation of liver lipids by inhibiting the autophagy level of hepatocytes 8. Therefore, will the down-regulation of the *ApoM* gene disrupt the balance of liver metabolism and function? Will it participate in the occurrence and development of liver cancer? At least so far, the evidence is insufficient.

Here, we first prove whether ApoM is associated with primary liver cancer. Analyzing 50 matched samples of primary liver cancer in the TCGA database, the expression level of ApoM in cancer tissues was lower than that in paracarcinoma tissues (Fig. 1A). Use the collected 23 cases of primary liver cancer paired samples for verification (Fig. 1B). The expression of ApoM in cancer tissues and adjacent tissues is related to age (<50, *P*=0.010), gender (male, *P*=0.012), tumor size (*P*=0.020), necrosis (*P*=0.024) and liver cirrhosis (*P*=0.028), factors related to poor differentiation (*P*=0.0010) (Table 1). The results are consistent with the database analysis results. The expression level of ApoM in the mouse liver cancer cell line was significantly lower than that in the normal liver cell line AML12 (Fig. 1C). Finally, in the liver cancer mouse model, the expression level of ApoM in the cancer tissue was also significantly lower than that in the paracarcinoma tissues (Fig. 7B). These evidences make us believe that ApoM may be a potential protective factor to inhibit the occurrence and development of primary liver cancer.

Unreserved rapid growth is one of the characteristics of tumor cells, and it is also the highlight of increased cell proliferation activity. Edu cell proliferation experiments showed that the proliferation activity in the knockout group increased significantly. Although it was not statistically significant, the proliferation activity in the overexpression group decreased (Fig. 2E, 2F). In order to further verify the experimental results at the *ex vivo* level, we subcutaneously injected the control group cells and the constructed knockout group cells into nude mice and observed the tumor formation rates of the two. We found that axillary lumps showed up faster in the knockout group of nude mice and were significantly larger than those in the control group of nude mice on the 16th day (Fig. 3A, 3C). Accordingly, we concluded that the deletion of the *ApoM* gene promotes the proliferation of the mouse liver cancer cells Hepa1-6. Cell cycle experiments further showed that the proportion of knockout group cells decreased in the G0/G1 phase, and the proportion in the S+G2/M phase increased, while the overexpression group was blocked in the G0/G1 phase (Fig. 3D, 3E).

Apoptosis is a programmed cell death mode regulated by genes. It is an effective way to eliminate senescence, damage, and precancerous cells in the human body 25, and down-regulation of apoptosis is one of the reasons for the excessive proliferation of tumor cells 26. In order to explore whether the level of *ApoM* gene expression affects the level of apoptosis of liver cancer cells, we used flow cytometry to detect the level of apoptosis in the knockout group and overexpression group induced by cisplatin. Different sampling times were introduced to reduce errors caused by transfection efficiency and expression efficiency. The results showed that the knock-out group had a lower level of apoptosis than the control group, while the overexpression group displayed the opposite trend (Fig. 4A). In addition, the WB results further supported the above results. The knockout group down-regulated (and the over-expression group up-regulated) the level of activity in the mitochondrial apoptosis pathway (Fig. 5A, 5B).

The growth status of tumor cells is certainly important, but more ominous is the ability of tumor cells to spread from the original site to surrounding tissues and even to metastasize further away. From the perspective of liver cancer, about 60% of patients' exhibit metastasis at the time of treatment, and even throughout active treatment there will still be a high recurrence rate 27. Inhibiting the migration and invasion of cancer cells is an important prerequisite for improving postoperative survival and ensuring a good prognosis. Unfortunately, we observed that knockout of the* ApoM* gene increased the migration and invasion capabilities of mouse liver cancer cells, while overexpression did not (Fig. 6).

In summary, ApoM plays a role in inhibiting the development of liver cancer cells as shown in proliferation, apoptosis, migration, and invasion related experiments. Does ApoM also play a role in the development of liver cancer? We used WT mice as the control group and* ApoM* knockout mice as the experimental group. We used N-nitrosodiethylamine to induce primary liver cancer, and observed whether the level of *ApoM* gene expression affected the occurrence of primary liver cancer. As expected, tumors appeared earlier in the livers of knockout mice (Fig. 7A, 7H). In addition, we also observed that when N-nitrosodiethylamine was induced for 12 weeks, the wet weight of the liver of the knockout mice increased the most (Fig. 7C), while the weight of knockout mice decreased significantly after 12 weeks (Fig. 7D). We also found that the level of apoptosis in WT mice was higher than that in* ApoM^-/-^*mice (Fig. 7G). The survival time of *ApoM^-/-^* mice was shorter than that of WT mice (Fig. 7I).

Obviously, in both *in vitro* and *in vivo* experiments, ApoM plays a role in inhibiting the occurrence and development of primary liver cancer. Here, we have obtained the corresponding results by regulating the expression level of the *ApoM* gene. In view of the increasing importance of the liver lipid metabolism environment in malignant liver disease, we initially took the influence of ApoM on liver lipid metabolism as a clue and entry point to explore the correlation between ApoM and liver cancer. And we also found that the down-regulation of the expression level of *ApoM* gene can lead to impaired liver function (Fig. 7E, 7F). The damaged liver will inevitably lead to a decrease in ApoM expression. Will this form a vicious circle? Therefore, in future work, we will further explore the specific mechanism that enables ApoM to affect the occurrence and development of liver cancer by regulating lipid metabolism. This will significantly improve our knowledge of the overall mechanism of primary liver cancer and enable us to find potential prevention and treatment targets.

## Figures and Tables

**Table 1:** Clinicopathological analysis of cancer tissues and adjacent tissues in 23 patients with liver cancer (SD)

	Paracancerous	Carcinoma	*P* value
**Age**			
≥50	99.34 (13.25)	91.25 (12.79)	0.052
<50	110.88 (11.04)	92.46 (4.62)	0.010
**Gender**			
Male	102.90 (13.05)	91.97 (12.75)	0.012
Female	97.24 (15.54)	89.68 (4.76)	0.241
**Tumor size**			
Y	101.27 (17.67)	92.39 (11.69)	0.020
N	105.68 (15.54)	83.81 (7.07)	0.071
**Necrosis**			
Y	106.29 (12.81)	90.27 (13.30)	0.024
N	99.32 (13.56)	92.14 (10.80)	0.085
**Sclerosis**			
Y	101.47 (12.16)	92.08 (10.21)	0.028
N	101.96 (15.08)	90.95 (12.96)	0.065
**Levels of differentiation**			
Low	108.54 (11.65)	87.51 (8.89)	0.001
Middle	97.80 (13.98)	96.56 (14.03)	0.811
High	98.44 (13.37)	90.93 (10.73)	0.245
